# Comparison of Test Setups for the Experimental Evaluation of the Primary Fixation Stability of Acetabular Cups

**DOI:** 10.3390/ma13183982

**Published:** 2020-09-09

**Authors:** Christian Schulze, Danny Vogel, Sina Mallow, Rainer Bader

**Affiliations:** Biomechanics and Implant Technology Research Laboratory, Department of Orthopaedics, University Medicine Rostock, Doberaner Straße 142, 18057 Rostock, Germany; danny.vogel@med.uni-rostock.de (D.V.); sina.mallow@uni-rostock.de (S.M.); rainer.bader@med.uni-rostock.de (R.B.)

**Keywords:** acetabular cup, acetabular defect, primary stability, lever-out test, edge-load test, total hip arthroplasty

## Abstract

Sufficient primary fixation stability is the basis for the osseointegration of cementless acetabular cups. Several test methods have been established for determining the tilting moment of acetabular press-fit cups, which is a measure for their primary fixation stability. The central aim of this experimental study was to show the differences between the commonly used lever-out test method (Method 1) and the edge-load test method (Method 2) in which the cup insert is axially loaded (1 kN) during the tilting process with respect to the parameters, tilting moment, and interface stiffness. Therefore, using a biomechanical cup block model, a press-fit cup design with a macro-structured surface was pushed into three cavity types (intact, moderate superior defect, and two-point-pinching cavity) made of 15 pcf and 30 pcf polyurethane foam blocks (*n* = 3 per cavity and foam density combination), respectively. Subsequently, the acetabular cup was disassembled from the three artificial bone cavities using the lever-out and the edge-load test method. Tilting moments determined with Method 1 ranged from 2.72 ± 0.29 Nm to 49.08 ± 1.50 Nm, and with Method 2, they ranged from 41.40 ± 1.05 Nm to 112.86 ± 5.29 Nm. In Method 2, larger areas of abrasion were observed in the artificial bone cavity compared to Method 1. This indicates increased shear forces at the implant–bone interface in the former method. In conclusion, Method 1 simulates the technique used by orthopedic surgeons to assess the correct fit of the trial cup, while Method 2 simulates the tilting of the cup in the acetabular bone cavity under in situ loading with the hip resultant force.

## 1. Introduction

In total hip arthroplasty, for the cementless fixation of acetabular cups, adjacent bone stock with sufficient elasticity [1], precise milling of the acetabulum [2,3,4,5,6,7,8,9,10,11], and sufficient overlap of the cup with the acetabulum [3] are required for generating the press-fit fixation. Consequently, the primary fixation stability of the implant takes place in the form of a friction-engaged connection between the implant and the acetabular bone stock. A large area of frictional contact between the implant and the bone promotes osseointegration of the implant and leads to good long-term stability [12,13,14]. The frictional contact is generated in the equatorial region of the acetabular cup [15,16]. In order to ensure the best possible bone ingrowth of cementless acetabular cups, the primary stability of press-fit cups has been experimentally investigated and characterized in recent decades. Therefore, various experimental models were developed to determine the fixation stability [2,17,18,19,20,21] and micromotion [15,22,23,24] as well as implant migration as a measure for the primary stability and to determine the deformation behavior of acetabular cups [25,26,27]. Determination of the primary fixation stability of press-fit acetabular cups has been performed predominantly using the lever-out method [1,2,28,29,30]. In this method, the press-fit cups are levered out of the milled acetabular cavity, primarily in artificial bone substitutes, through a lever arm screwed into the pole of the acetabular cup [1,2,28,30]. This test method mimics the method employed intraoperatively to assess the initial stability of the trial acetabular cup [15,31], which has the same shape as the final acetabular cup but does not have a structured surface resulting in reduced press-fit. To experimentally estimate the performance of an acetabular cup in the pelvic bone of a human, this biomechanical load-to-failure model was partly implemented to the human pelvis in cadaver studies [32,33,34], and the primary stability was characterized as a function of the individual bone material properties and the acetabular cup size. The material properties of human pelvic bone commonly show high inter-specimen variability between different individuals. Due to the reasons of reproducibility and comparability of the experimental results and the limited availability of human donors, polymer foams are predominantly used as bone substitute materials [1,2,16,19,22,23,25,27,30,35,36,37,38]. In the lever-out method, the center of rotation (CoR) at which the acetabular cup is levered out of the artificial bone block is located in the interface between the acetabular cup and the acetabular cavity in the loading direction. This load-to-failure test neglects the occurrence of moments in the acetabular cup caused by the hip resultant force [39] and the friction moments between the articulating cup and the femoral head [40,41] during the gait cycle. Therefore, the central aim of the present study was to show the difference between the lever-out test method and a test method, which involves a tilting load on the edge of the cementless fixed acetabular cup [42,43,44] when an axial load is simultaneously introduced into the liner of the acetabular cup on a biomechanical cup-block model. Furthermore, the influence of the experimental boundary conditions such as the cavity shape (defect models) and artificial bone densities (15 pcf and 30 pcf) on primary stability parameters of the acetabular cup was determined. Hence, the tilting moment and interface stiffness obtained from both test methods and the tilting mechanism of both the methods were characterized.

## 2. Materials and Methods

### 2.1. Preparation of the Specimens

**Figure 1:**
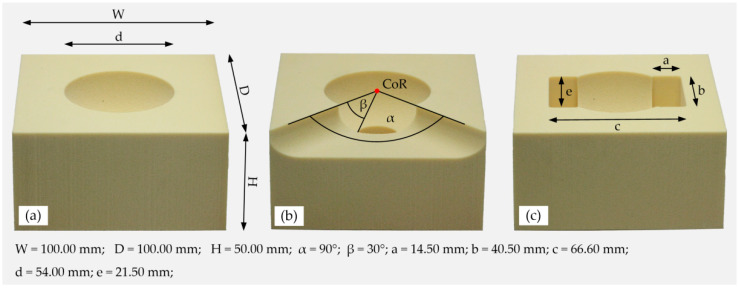
Polyurethane (PU) foam cavities for the experimental tests and the ideal geometry parameters associated with different cavity types. Three different cavity types were considered: (**a**) intact cavity (Intact); (**b**) a moderate superior acetabular defect (Defect 1), and (**c**) a two-point pinching cavity model (Defect 2).

For the experimental investigation of the influence of the tilting method on the primary stability of a cementless acetabular cup, a biomechanical cup-block model was used. For this purpose, polyurethane (PU) foam blocks (Sawbones^®^, Malmö, Sweden) with densities of 15 pcf (0.24 g/cm^3^, E = 173 MPa) and 30 pcf (0.48 g/cm^3^, E = 592 MPa) were used as artificial bone materials to represent the acetabular cavity. Three types of cavities were considered for the tests: the intact cavity (Intact), a moderate superior acetabular defect (Defect 1), according to our previous study [1], and a two-point pinching cavity model (Defect 2), according to Jin et al. [25] (Figure 1). Defect 1 (D1) represents a superior acetabular defect in which 50% (α = 90°) of the superior acetabular rim (defect width) and 33% (β = 30°) of the mediolateral wall (defect depth) are lost. This defect was identified as the maximum defect extent to which the anchoring of a primary acetabular cup seems to be possible [1]. Furthermore, the cavity of Defect 2 (D2) was adapted to the shape of the acetabular cup, according to Meding et al. [27]. The two-point pinching model was assumed to be the worst case of clamping of an acetabular cup in terms of implant deformation and is therefore often used [25,26]. Three identical blocks (*n* = 3) were manufactured by means of computer numerically controlled (CNC) milling for every combination of foam density and defect type. The cavities with an inside diameter of 54 mm were chosen according to the surgical technique of the utilized acetabular cup, which resulted in a nominal diametric press-fit of 2 mm. For the tests, the Allofit^®^ press-fit cup (54/JJ, Zimmer Inc., Warsaw, IN, USA) with a nominal outer diameter of 56 mm was used.

According to the measurements of Weissman et al. [35], the diameter of the acetabular cup was measured using a non-contact measuring microscope (Mitutoyo—QVE-200 Pro, Mitutoyo Corporation, Kawasaki, Japan). In contrast, the cavity diameters were determined using a caliper gauge in three directions in the artificial bone cavity (Table 1).

### 2.2. Experimental Setup

#### 2.2.1. Push-In Procedure

Before testing the lever-out method (Method 1) and the edge-load method (Method 2), the acetabular cup was pushed into the artificial bone cavities in the same manner. During the push-in process, the PU foam blocks were mounted on a transverse load bearing support in order to reduce any lateral forces, and the acetabular cups were concentrically aligned with the cavities. The acetabular cup insertion was conducted under displacement-controlled conditions using a universal testing machine (Zwick/Roell Z050, Zwick GmbH & Co. KG, Ulm, Germany) at a crosshead velocity of 5 mm/min. Simultaneously, the insertion force was recorded until the acetabular cup was seated in the predefined optimum position as per the surgical technique [1]. The implant position was optically evaluated, and the cup was considered ideally placed when the upper edge of the macroscopic surface structure of the acetabular cup was at the block surface level. Subsequently, the acetabular cup was either levered (Method 1) or tilted out (Method 2) of the cavities using a universal testing machine (Zwick/Roell Z050), and the lever-out moments as well as the interface stiffness were evaluated in dependency of the chosen test method.

#### 2.2.2. Lever-Out Method

**Figure 2:**
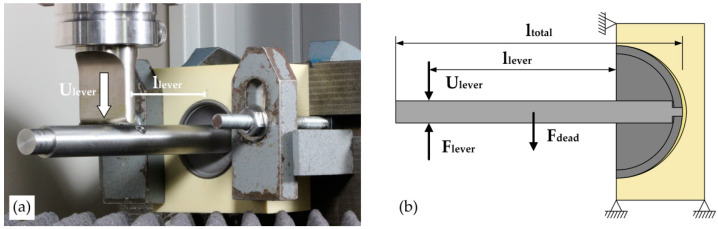
Test method 1—Displacement-controlled lever-out test. (**a**) Experimental test setup for the lever-out test. (**b**) A schematic view of the lever-out test. The parameters required calculating the lever-out moment—the effective lever arm length (l_lever_), the applied displacement (U_lever_), and the measured ultimate lever-out force (F_lever_) have been shown. The intrinsic moment resulting from the parameters—the deadweight of the lever shaft (F_dead_) and the total length of the lever shaft (l_total_)—were also taken into account (Formula (1)).

In this test method, a shaft screwed into the pole of the acetabular cup was used to lever the implant out of the PU foam cavities (Figure 2). The displacement (U_lever_) required to lever the acetabular cup out was perpendicularly applied at a crosshead velocity of 5 mm/min to the axis of the lever shaft in the direction of the defect using a universal testing machine (Zwick/Roell Z050). Simultaneously, the reaction force was recorded, and the lever-out moment (M_lever_) was calculated on the basis of the ultimate lever-out force (F_lever_) and the effective lever arm length (l_lever_). In addition, the moment (M_dead_) generated by the deadweight of the lever-out shaft (F_dead_), which acts at the half the length of the lever-out shaft (l_total_), was taken into account by adding it to the measured lever-out moment (Formula (1)):

M_lever_ = (F_lever_ × l_lever_) + (F_dead_ × 1/2 × l_total_). (1)

A detailed description of the lever-out test method was provided in our previous study [1]. The lever-out test was performed *n* = 3 for each combination comprising of different cavity types and foam densities.

#### 2.2.3. Edge-Load Method

**Figure 3:**
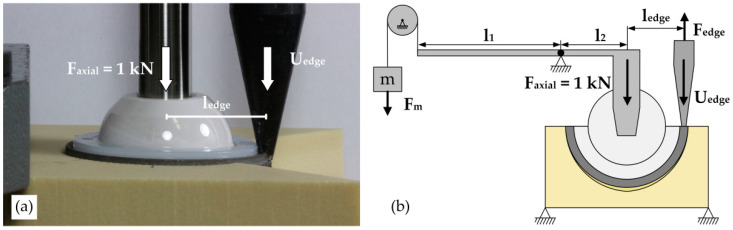
Test Method 2. (**a**) Experimental test setup for the displacement-controlled edge load test. (**b**) A schematic view of the edge-load test. By means of a lever construction (l_1_ and l_2_) in combination with a deflection roller, the weight (F_m_) of the mass (m) introduced an axial force F_axial_ = 1 kN into the liner of the acetabular cup. The tangential displacement of the cup edge (U_edge_) to the implant–block interface counteracted the resistance moment (M_edge_). The moment (M_edge_) resulted from the distance between the axial force (F_axial_) and the reaction force (F_edge_), which counteracted the displacement of the cup edge.

In contrast to the lever-out method (Method 1), in the edge-load method (Method 2), the acetabular cup is tilted out of the block by loading the edge of the implant, while a force is axially introduced into the liner of the acetabular cup, according to Zietz et al. and Souffrant et al. [37,38]. For this purpose, an axial load (F_axial_) of 1 kN following the hip resultant force [32,37,38], which corresponds to the loading of the acetabular cup in a bipedal stand or slow walk, was applied to the liner (Durasul PE Inlay, Zimmer Inc., Warsaw, IN, USA) using an external lever mechanism with the lever law (Figure 3).

Here, the axial load (F_axial_) multiplied by the lever arm (l_axial_) is equal to the weight force (F_mass_) applied on the opposite side of the lever mechanism multiplied by the lever arm of the mass (l_mass_):

F_mass_ × l_mass_ = F_axial_ × l_axial_. (2)

The external lever mechanism was connected to the liner of the acetabular cup through an alumina-toughened zirconia ceramic ball head with a diameter D = 36 mm (36(M), 12/14 taper, Ceramys^®^, Mathys Orthopaedie GmbH, Mörsdorf, Germany) mounted on the pivot pin at the end of the lever arm (l_axial_). The articulating surfaces between the ceramic head and liner were lubricated with a bovine serum (20 g/L protein concentration, 1.85 g/L sodium azide (NaN3) and 5.85 g/L ethylene diamine tetraacetic, Biochrom GmbH, Berlin, Germany) to reduce the shear stresses and thereby the friction moments in the head–liner interface. Subsequently, the acetabular cup was tilted in the direction of the cavity by applying a linear displacement on the edge of the cup, which acts tangential to the implant–cavity interface. Therefore, the resulting rotational movement of the acetabular cup was guided by the artificial bone cavity shape, and thus, a real moment was assumed to be generated at the CoR of the outer surface of the acetabular cup. The displacement (U_edge_) is vertically applied on the edge of the cup under displacement-controlled conditions at a crosshead velocity of 5 mm/min by the pin attached to the universal testing machine (Zwick/Roell Z050) (Figure 3). During the tilting process, the reaction force was recorded. By multiplying the ultimate tilting force (F_edge_) by the distance between the CoR of the acetabular cup and the acetabular cup edge (l_edge_), the tilting moment (M_tilt_) was calculated (Formula (3)):

M_tilt_ = (F_edge_ × l_edge_). (3)

The edge-load test was performed *n* = 3 for each combination comprising different cavity types and foam densities.

#### 2.2.4. Analyzed Parameters

**Figure 4:**
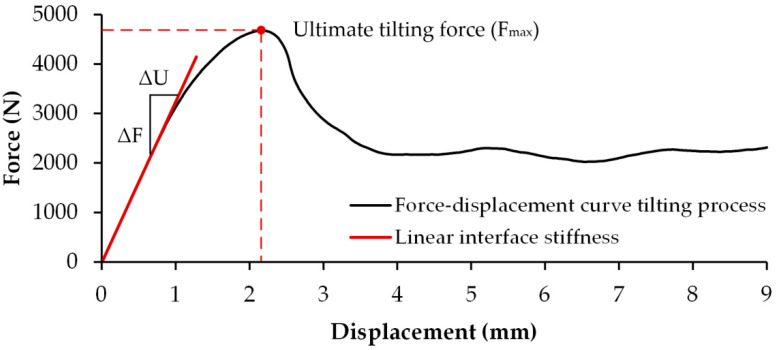
Typical force–displacement curve of the acetabular cup tilt, including the different fixation stability parameters: ultimate tilting force (F_max_) and interface stiffness (k_inter_ = ΔF/ΔU).

Parameters such as insertion force F_in_, moment M, and interface stiffness k determined with both test methods were used to characterize the primary stability of the acetabular cup and determine the influence of the test method on the measured primary implant stability. In both test methods, the maximum tilting force (F_max_) during the tilting process was used to calculate the parameter moment M (Figure 4). In the region of the rise at the beginning of the force–displacement curve, the interface stiffness was determined by a linear regression (Figure 4). For this purpose, the maximum slope of the force–displacement curve was determined over four consecutive pairs of values, which were subsequently used for the regression analysis. Care was taken to ensure that a coefficient of determination of at least R^2^ = 0.999 was achieved. The slope (m) of the resulting linear regression function (f_reg_) is described by the relationship between force change (ΔF) and change of displacement (ΔU) (Formula (4)):

f_reg_ = (ΔF/ΔU) × x + n. (4)

The linear interface stiffness between the acetabular cup and the PU foam blocks can be directly obtained from the slope of the regression function.

Based on the averaged force–displacement curves, the test methods were characterized as functions of cavity type and foam density. Additionally, after disassembling the acetabular cups from the foam cavities using Method 1 and Method 2, the cavity surfaces were exemplarily compared and qualitatively assessed for analyzing the damage to the surface of the artificial bone cavity.

#### 2.2.5. Statistical Analysis

All the data given in the diagrams are expressed as mean values ± standard deviation (SD). All the analyses of the statistical significance were performed using SPSS V25.0 (SPSS^®^ Inc., Chicago, IL, USA). All of the experimentally determined parameters were checked for compliance with the requirements (standard normal distribution and homogeneity of the variances) of the one-way analysis of variances test (ANOVA). The parameter insertion force fulfilled the requirements and was examined using ANOVA and Bonferroni post-hoc test for establishing statistically significant differences between the different methods. The parameters’ lever-out moment and interface stiffness violate the criteria of variance homogeneity. Therefore, Welch’s ANOVA test with a post-hoc Games–Howell test was performed to determine the significant differences between the different methods and defects. A level of *p* < 0.05 was considered statistically significant.

## 3. Results

### 3.1. Insertion Forces

**Figure 5:**
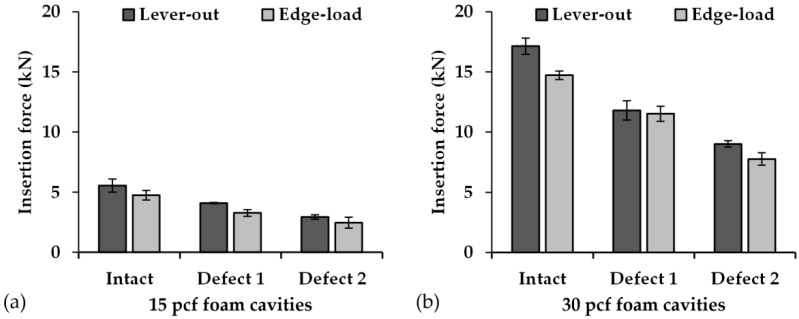
Comparison of the forces of the acetabular cup insertion for both the tilting methods: (**a**) for the PU foam with a density of 15 pcf and (**b**) for the PU foam with a density of 30 pcf. The insertion forces are presented as mean values (*n* = 3), and the error bars represent SD.

When acetabular cups were pushed into the cavities prepared for Method 1, the insertion forces in the range from 2.93 ± 0.20 kN (M1_15_D2) to 17.14 ± 0.68 kN (M1_30_I) were determined. The cavities prepared for test Method 2 showed insertion forces within the range of 2.46 ± 0.46 kN (M2_15_D2) to 14.72 ± 0.36 kN (M2_30_I). When comparing the push-in test for Method 1 and Method 2, no statistically significant differences could be found between the cavities with the same defect extent and foam density (Figure 5). The artificial bone cavities with a density of 30 pcf exhibited an average of 317 ± 41% higher insertion forces, compared to the artificial bone cavities with a density of 15 pcf. Thus, the amount of insertion force declines with the decreasing foam density. Additionally, a decrease of the insertion force can also be observed with increasing defect size.

### 3.2. Lever-Out and Tilting Moments

**Figure 6:**
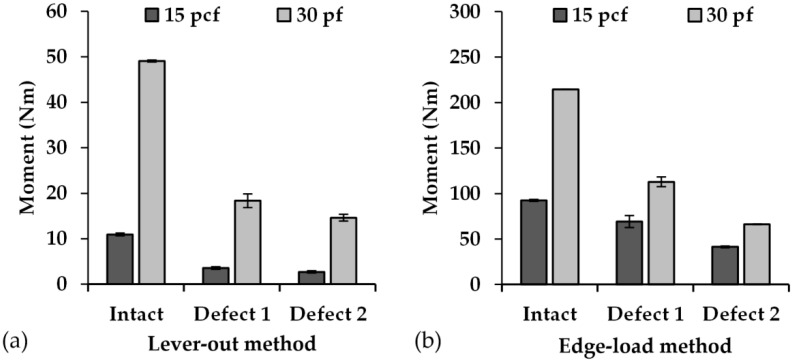
Comparisons of the determined moments of the acetabular cups with regard to the combination of cavity type and foam density for (**a**) the lever-out test method (Method 1) and (**b**) the edge-load test method (Method 2). The tilting moments are presented as mean value (*n* = 3), and the error bars represent the SD. The intact cavity made of 30 pcf foam density tested with Method 2 is the only exception, where only one cup could be tested. For reasons of better visibility, the scaling of both graphs was adjusted to the determined values.

When lever-out moments were measured using Method 1, the values were within the range of 2.72 ± 0.29 Nm (M1_15_D2) to 49.08 ± 1.50 Nm (M1_30_I). In contrast, values of tilting moments within the range of 41.40 ± 1.05 Nm (M2_15_D2) to 112.86 ± 5.29 Nm (M2_30_D1) were determined using Method 2 (Figure 6). With Method 2, the maximum tilting moment of 214.37 Nm was determined at the intact cavity and foam density of 30 pcf. However, only one cup could be evaluated, as plastic deformation of the implant material occurred in the other two cups at the contact point between the loading pin and the cup rim due to the punctual loading of the cup edge.

Upon increasing the defect extent, the measured moments decreased in both of the test methods due to the reduction of the inner surface of the cavity and a decrease in the implant–block interface. The moment determined with Method 1 reduced by about 78% with intact cavity, by about 81% with Defect 1, and by about 80% with Defect 2 when using 15 pcf foam density compared to 30 pcf. Those determined with Method 2 decreased by about 57% with intact cavity, by about 40% with Defect 1, and by about 37% with Defect 2. On average, the tilting moment increased by 343 ± 160% when the foam density of the artificial bone was doubled; however, this influence decreased in Method 2 with an increase in the defect extent. Moreover, an increase in the defect extent from Intact to Defect 1 and from Intact to Defect 2 caused an average reduction of the lever-out moment in Method 1 of approximately 67% and 75% for the foam cavities with 15 pcf density as well as by about 63% and 70% for the foam cavities with 30 pcf density, respectively. When using test Method 2, the interface stiffness was reduced from Intact to Defect 1 by about 25% and from Intact to Defect 2 by about 55% for the foam cavities with 15 pcf density as well as by about 46% and 69% for the foam cavities with 30 pcf density. There were statistically significant differences in both of the test methods for the same defect foam density combinations in terms of the determined moments. The comparison between the combinations of defect type and foam density within the test methods also exhibited significance differences (Table 2).

### 3.3. Interface Stiffness

**Figure 7:**
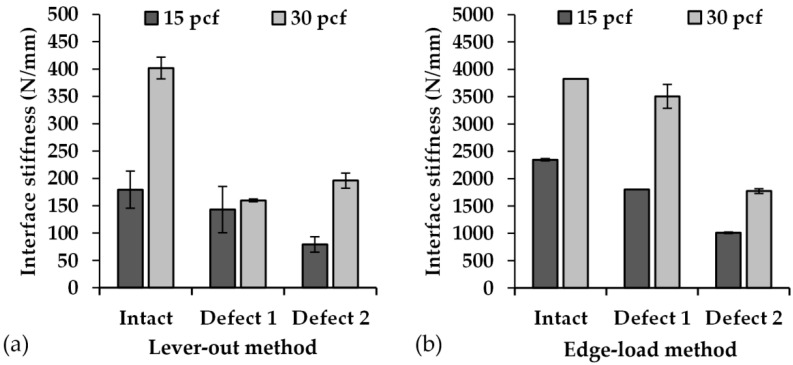
Comparisons of the determined interface stiffness of the acetabular cups with regard to a combination of cavity type and foam density for (**a**) lever-out test method (Method 1) and (**b**) edge-load test method (Method 2). The interface stiffness are presented as mean values (*n* = 3), and the error bars represent the SD. The intact cavity made of 30 pcf foam density tested with Method 2 is the only exception, where only one cup could be tested. For reasons of better visibility, the scaling of both graphs was adjusted to the determined values.

In both test methods, the linear slope at the beginning of the force–displacement curve in the tilting process was used to determine the interface stiffness. Thus, the interface stiffness determined using Method 1 ranged from 79.07 ± 14.21 N/mm (M1_15_D2) to 401.88 ± 19.74 N/mm (M1_30_I). In contrast, the interface stiffness determined with Method 2 ranged from 1012.91 ± 73.37 N/mm (M2_15_D2) to 3504.77 ± 220.32 N/mm (M2_30_D1) (Figure 7). The intact cavity with a density of 30 pcf could not be taken into account as plastic deformation of the cup edge occurred in the case of these intact cavities. Therefore, the interface stiffness (3823.5 N/mm) of only one of the three used acetabular cups could be evaluated.

The interface stiffness determined with Method 1 reduced by about 55% with intact cavity, by about 11% with Defect 1, and by about 60% with Defect 2 when using 15 pcf foam density compared to 30 pcf foam density. Those determined with Method 2 decreased by about 39% with intact cavity, by about 49% with Defect 1, and by about 43% with Defect 2. Moreover, an increase in the defect extent from Intact to Defect 1 and from Intact to Defect 2 caused an average reduction of the interface stiffness determined with Method 1 of about 20% and 56% for the foam cavities with 15 pcf density as well as 60% and 51% for the foam cavities with 30 pcf density, respectively. When using test Method 2, the interface stiffness was reduced from Intact to Defect 1 by about 23% and from Intact to Defect 2 by about 57% for the foam cavities with 15 pcf density as well as by about 8% and 53% for the foam cavities with 30 pcf density. For the artificial bone with the same defect extent and the same density, significant differences were found between Method 1 and Method 2 for the parameter interface stiffness. The comparison between the combinations of defect type and foam density within the test methods also exhibited significance differences (Table 3).

### 3.4. Qualitative Characterization of the Two Test Methods

**Figure 8:**
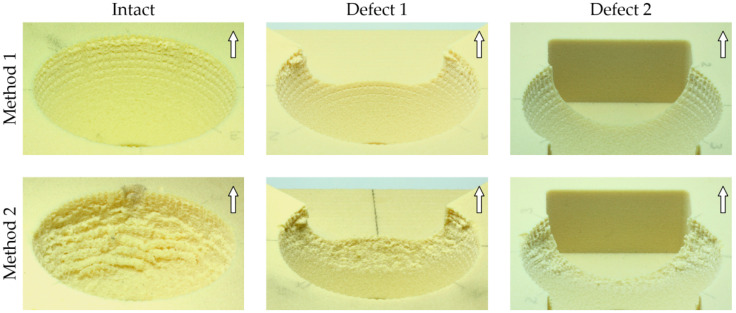
A visual comparison of the cavity surfaces after disassembling the acetabular cups from the PU foam cavities (30 pcf) with Method 1 and Method 2. The arrows in the upper right corner of the figures represent the direction of acetabular cup tilting (superior). In all the cavities tested with Method 1, clear impressions of the barb-like surface structure of the acetabular cup, caused by cup insertion, were present. The small areas where shear stress was detected were located in the direction of acetabular cup tilting (superior). In the acetabular cups tested with Method 2, extensive abrasion of the foam structure was present in the direction of acetabular cup tilting, indicating the occurrence of higher shear stress in the implant–bone interface.

After testing with Method 1 and Method 2, the optical comparison of the cavities demonstrated clear differences at the surfaces of the artificial bone cavities (Figure 8). With Method 1, impressions of the macroscopic surface structure on the PU foam were visible, which were clearly separate from one another. In the region of equatorial clamping of the acetabular cup, slight abrasive damage of the foam could be observed, indicating a slight occurrence of shear stress in the implant–cavity interface during the lever-out process. Conversely, after tilting the cups using Method 2, impressions of the barb-like macrostructure could only be determined at the level of the artificial bone cavity rim. However, extensive foam surface damage was visible in the cavities, which was caused by high shear stresses in the implant–bone interface.

### 3.5. Force–Displacement Behavior

**Figure 9:**
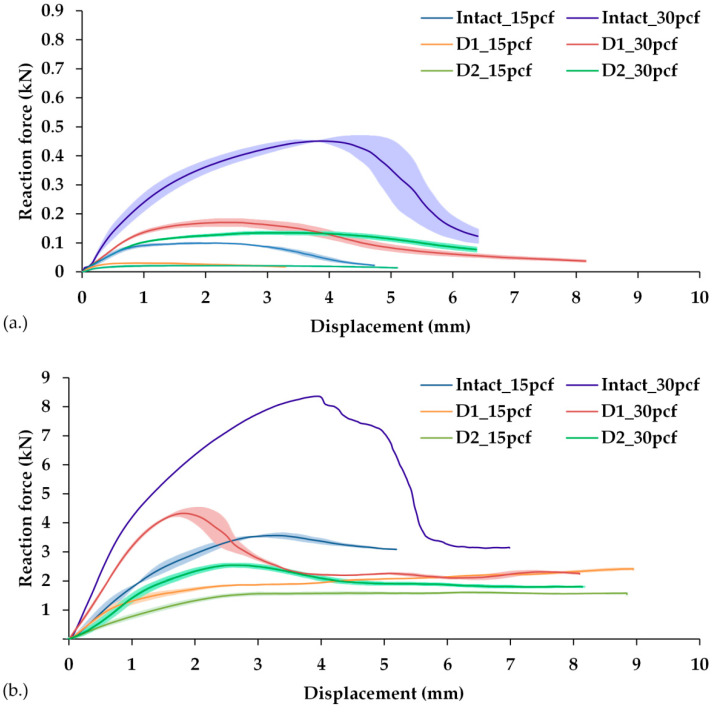
Force–displacement curves determined using Method 1 (**a**) and Method 2 (**b**) for tilting the acetabular cups from all the cavity types at 15 pcf and 30 pcf foam densities. For qualitative comparison, the curves are represented by their mean values (*n* = 3) for each cavity type-foam density combination, and the colored area around the curve represents the SD. The only exception in the force–displacement curves is for the intact cavity in the foam block of 30 pcf density in Method 2. Moreover, due to the occurrence of plastic deformation at the rim of the acetabular cup, only one cup could be tested. For reasons of better visibility, the scaling of both graphs was adjusted to the determined values.

With both the methods, the tilting process could be divided into three characteristic stages: (1) elastic deformation in the implant–block interface (approximate linear elastic range); (2) elastic–plastic deformation (the range of maximum fixation strength); (3) final loosening of the implant. The range of the linear slope of the force–displacement curves described the interface stiffness, which was usually less pronounced in Method 1 (lever-out method) than in Method 2 (edge-load method). Furthermore, Method 2 exhibited the maxima of reaction force during the tests, which was more pronounced than that produced by Method 1. This difference in the tilting characteristics increased with the rising foam density. After reaching the maximum force, the force in Method 1 steadily decreased until the implant–block interface was completely loose. The cup was completely disassembled from the artificial bone cavity. In contrast, tilting with Method 2 took place tangentially in the direction of the cavity. Moreover, the cup–block interface was not disintegrated, and the rotational movement of the cup was guided by the axially introduced force and the shape of the artificial bone cavity. After exceeding the maximum force, the reaction force decreased to a value that remained almost constant, while further displacement of the cup in the direction of the cavity took place. This effect occurred independently of the density of the used foam. The force–displacement curves for cavities with the same defect but different density approaches had approximately the same values in the curves (Figure 9), which confirmed this observation.

## 4. Discussion

The prerequisite for sufficient bone integration of cementless acetabular press-fit cups is the primary stable fixation of the implant in the acetabular bone cavity. In order to characterize and quantify the mechanism of primary implant stability, different experimental test methods have been established. One of these methods that entail the acetabular cup being levered out of the bone cavity with a lever arm screwed into the pole of the cup has been established as the most commonly used method for determining fixation stability in terms of the final failure of the acetabular cup. However, this method disregards the joint resultant force [39] in vivo caused by muscle and soft tissue tension [45,46] and loading of the acetabular cup rim tangential to the implant–bone interface in the case of an impingement event [47,48,49,50]. Therefore, the central purpose of our present experimental study was to characterize and show the differences between the lever-out method and the edge-load method in which the load for tilting is applied on the edge of the acetabular cup, while a force is simultaneously introduced into the acetabular cup following the hip resultant force. Furthermore, three types of cavities were considered for the tests: the intact cavity (Intact), a moderate superior acetabular defect (Defect 1), and a two-point pinching cavity model (Defect 2). The highest insertion forces and tilting moments were observed in the intact cavity for PU foam with 15 pcf and 30 pcf densities. Doubling the foam density led to a tripling of the insertion force and, on average, to a quadrupling of the tilting moment. Assuming that the compression modulus is the main contributor in generating the radial forces for acetabular cup clamping, this could also be explained by the increase in the compressive modulus from 128 MPa for the PU foam with 15 pcf density to 391 MPa for the PU foam with 30 pcf density [30]. This correlation was observed in a previous study [30] and could be observed in the present study, especially in the case where acetabular cups were inserted in the intact cavity. From the surgical perspective, high seating forces, as determined for the 30 pcf foam, might be problematic for the insertion of the acetabular cup [10,28] due to the risk of intraoperative periprosthetic fractures [3]. In this case, the amount of press-fit would be adapted according to the stiffer bone situation with additional reaming of the acetabulum [51].

The insertion process plays an important role in the primary stability of the cementless acetabular cups. If the acetabular cup is inserted with the same maximum force in force-controlled conditions [20], the final cup position is set according to the surface roughness as a balance of the friction forces, which depend on the friction coefficient and the normal forces generated by the press-fit and the applied seating force on the artificial bone. This results in different cup-seating depths, but almost identical tilting moments [20]. However, if the cementless acetabular cups are inserted in displacement-controlled conditions until the optimum seating position is reached, a comparison can be made between the two methods with regard to the tilting stability in each, with a consideration of the press-fit. When determining the primary stability parameters, the used press-fit cup design showed significant differences between Method 1 (lever-out method) and Method 2 (edge-load method). The micro surface roughness of the acetabular cup used in this study promotes the frictional connection between the acetabular cavity and the implant, which is required to generate the primary fixation stability. However, the influence of different surface structures, such as porous coatings [32], different rough blasting [20,37,42], and varying macrostructures [16] on the primary stability was not within the scope of the present study. The macroscopic barb-like surface structure of the acetabular cup used in this study led to an interlocking of the acetabular cup and the reamed cavity in the implant–bone interface [1]. However, it is also possible that the macro-spikes in the equatorial zone of the acetabular cup could negatively influence the primary stability through excessive plastic deformation of the foam [21].

The tilting moments and interface stiffness determined with the Method 2 were 869 ± 564% and 1216 ± 423% higher on average respectively, compared to Method 1. On the one hand, the higher tilting moments and interface stiffness in Method 2 could be explained by the displacement of the cup in the direction of the cavity tangential to the implant–bone interface and, on the other hand, by the fact that in Method 1, the cup was levered out of the cavity to a greater extent. This could lead to partial relief on the side of the cavity opposite to the lever-out direction in Method 1 and thus to lower moments and shear forces in the implant–block interface. Furthermore, in Method 2, the additional axial load introduction into the liner of the acetabular cup increased the forces acting tangentially in the interface, which can lead to increased shear stresses in the interface. Consequently, the tilting moment in Method 2 was additionally influenced more strongly by the macroscopic barb-like structure on the cup surface than in the Method 1. The axial load introduction was chosen according to the literature [43,52,53] and simulated the resultant hip force [37,52] during a bipedal stance or a slow walk [3,52].

A visual examination of the bone cavity surfaces after tilting with Method 1 and Method 2 revealed that there were clear traces in the equatorial anchoring zone with Method 1, as described for the other cup types [16,21,37]. These traces corresponded to the plastic deformation of the foam and were generated by inserting the acetabular cup. Method 2 showed significant abrasive damage from the equatorial to the polar region of the cavity, which indicated high shear forces and supported the assumption of increased shear stress in the implant–bone interface in Method 2. Due to the missing rim support in the superior direction in Defect 1, the stiffness in this direction altered, which meant that no or only reduced force could be generated in the inferior–superior axis for the cup fixation, and the force results in a reduction of the tilting moment in this direction. It was assumed that a four-point contact formed between the zones near the defect and the opposite contact zones in the cavity in the implant–bone interface at the level of the cup equator [1]. Additionally, a three-point contact has been described in the literature as the minimum criteria for anchoring of an acetabular cup in the presence of primary stability [54,55]. In Defect 2, the rim support was missing in the superior and the inferior direction, whereby the acetabular cup could only be clamped between the two points in the anterior–posterior axis. This represents the absolute worst situation in the case of anchoring a primary acetabular press-fit cup [25,27,56]. For interface stiffness, a significant difference between Defect 1 and Defect 2 was determined with Method 2 at both the foam densities (15 pcf and 30 pcf), which supported the assumption that the extent and the direction of the missing rim support relative to the direction of the cup tilt affect the interface stiffness. In Method 2, a reduction in foam density seemed to have less effect on the interface stiffness because the interface stiffness was superimposed by the axial load introduction in the acetabular cup during the tilting process. Method 1 indicated significant differences between the cavities with increasing defect extent only for 30 pcf foam density. Other studies determined moments with the lever-out method ranging from 1.13 to 55.5 Nm [1,2,16,17,18,19,20,21,28,30,35,36,57,58], whereby the moments determined with Method 1 (lever-out method) in our study ranged from 2.72 ± 0.29 Nm (M1_15_D2) to 49.08 ± 1.50 Nm (M1_30_Intact), which were in good agreement with the literature. Conversely, the tilting moments determined with Method 2 (edge-load method) ranged from 41.40 ± 1.05 Nm (M2_15_D2) to 112.86 ± 5.29 Nm (M2_30_D1). Due to the occurrence of plastic deformation of the rim of the two cups in the intact cavity and at 30 pcf foam density, the titling moment for only one acetabular cup (214.37 Nm) could be evaluated. Based on the assumption that the highest reaction forces during tilting of the acetabular cups can be expected at the rim in an intact cavity, these tests were performed at the end of the test series, first for 15 pcf density foams and afterwards for 30 pcf density foams. Thus, the influence of the plastic deformation of the acetabular cups on the other edge-load test results could be avoided. In the literature, tilting moments ranging from 1.1 to 83.7 Nm have been reported using the edge-load method [37,38,42,43,59,60,61,62]. By tilting six acetabular cup designs (56 mm, elliptical/hemispherical with three different surface porosities) from intact artificial bone cavities (0.22 g/cm^3^), Saleh et al. determined tilting moments between 56.3 ± 7.4 Nm and 76.2 ± 2.9 Nm with 2 mm press-fit [43]. Small et al. determined tilting moments ranging from 49.5 ± 9.9 Nm to 83.7 ± 10.1 Nm for four different cup designs (58 mm diameter) at 1 mm press-fit in intact PU foam cavities (15 pcf, 0.24 g/cm^3^ and 20 pcf, 0.32 g/cm^3^) [59]. Zietz et al. and Souffrant et al. reported tilting moments within the range of 57 ± 2 Nm to 74 ± 3 Nm while investigating the cup shape with simultaneous variation in the cup diameter (58 mm and 60 mm) and their influence on the primary stability [37,38]. In addition to the selected cup design, the tilting stability determined with the lever-out method as well as the edge-load method was significantly influenced by parameters such as the material of the acetabular cavity (human, polymeric foams) [16], density of the cavity material [30], cavity type (intact/moderate defects/large defects) [1], press-fit [15,21] and crosshead speed. The aforementioned parameters, which influence the primary stability, were varied or selected by the authors in the literature in accordance with the scientific questions they wished to answer. However, this leads to a wide range of tilting moments that were not directly comparable, which made it difficult to put them into context with the data provided by other studies. Our current results are in good agreement with the studies mentioned above [37,38,43,59] considering the axial load introduction of 1 kN into the liner of the acetabular cup. However, our biomechanical study contained some inherent limitations. With both test methods, the acetabular cup tilt could only be measured via the displacement transducer of the test machine crosshead and not with external transducers (e.g., linear variable differential transducers). Therefore, it was not possible to determine with sufficient accuracy the tilting of the acetabular cups corresponding to the critical micromotions (150 µm); thus, a direct link between micromotions and fixation stability, as shown in the literature [15,24], was not possible. A further limitation was that at the time of the investigation, the exact position of the CoR of the acetabular cup in relation to the external cup geometry and in relation to the CoR of the femoral head was not available due to missing construction data (CAD) of the acetabular cup. The artificial bone material used could only reproduce the properties of human bone with regard to the mechanical properties of a cancellous bone to a certain extent. Moreover, the surface roughness of the acetabular cup was not determined, wherein the influence of friction on the primary fixation stability, which can be caused by the different foam densities, could not be investigated. The observed abrasion of the cavity surface in the artificial bone was only used to identify the shear forces in the implant interface and might not be as pronounced in the native acetabulum due to the variant material properties of the native bone. However, artificial bone cavities were easily available, and they improve the repeatability of the experiments as a result of the consistency of their mechanical properties [63,64,65,66]. All of the cavity models used could only represent the anatomy of the native acetabulum to a limited extent [1].

Furthermore, the force introduced by the edge-load method is limited in terms of direction as well as magnitude, compared to the joint forces acting in vivo, as the force introduction was axial [37,38,67] and did not correspond to a loading scenario in a one-legged position [39]. However, the edge-load method with load introduction into the cup insert offers the possibility of testing the primary stability for different amounts and directions of the force introduction. The influence of the surface structures on the primary stability could also be tested in different loading scenarios.

## 5. Conclusions

The determination of the primary implant stability parameters, tilting moment, and interface stiffness, as well as the qualitative consideration of the force–displacement curves characterizing the tilting process of an acetabular cup and the visual examination of the bone cavity surfaces after the tests showed, as might be expected, great differences between the two test methods used (the lever-out method and the edge-load method). However, the fixation stability of one acetabular cup design could be investigated and characterized for both the test methods with respect to different properties of the primary fixation. Both test methods cannot be compared directly with each other due to the different failure mechanisms, but both methods tend to react to the change in foam density and cavity shape to the similar extent. The tilting moment and interface stiffness parameters determined with Method 1 were only influenced by the foam density and the cavity type, i.e., the stiffness of the cavity. In Method 2, the axial load introduction and displacement of the acetabular cup tangentially to the implant–block interface increases the influence of the outer surface structure of the implant on the primary stability parameters and thus superimposes the stiffness of the acetabular cavity. Both methods can be employed to determine the primary cup stability. The lever-out method is better suited to simulate the intraoperative situation of an unloaded acetabular cup. In contrast, the edge-load method with axial load introduction better reproduces the in vivo load situation of an acetabular cup triggered by resulting joint reaction forces and is therefore more suitable for investigating the design of acetabular cups and the surface properties. From our point of view, both test methods should be used in parallel to gain a better understanding of acetabular cup fixation during the development process.

## Figures and Tables

**Table 1:** Denotation and classification of the test sample groups based on the combination of test methods, foam densities, and cavity types, including the measured cavity diameter.

Denotation	Test Method	Foam Density (pcf)	Cavity Type	Measured Diameter (mm)		Press-Fit (mm)
				Cup	Cavity	
M1_15_I	Lever-out (Method 1)	15	Intact	55.30 ± 0.06	54.31 ± 0.07	0.99± 0.08
M1_15_D1			Defect 1		54.22 ± 0.07	1.07 ± 0.06
M1_15_D2			Defect 2		54.31 ± 0.05	0.98 ± 0.08
M1_30_I		30	Intact		54.11 ± 0.02	1.18 ± 0.07
M1_30_D1			Defect 1		54.07 ± 0.06	1.23 ± 0.10
M1_30_D2			Defect 2		54.02 ± 0.02	1.28 ± 0.07
M2_15_I	Edge-load (Method 2)	15	Intact		54.32 ± 0.08	0.97 ± 0.11
M2_15_D1			Defect 1		54.22 ± 0.04	1.07 ± 0.07
M2_15_D2			Defect 2		54.33 ± 0.05	0.96 ± 0.10
M2_30_I		30	Intact		54.12 ± 0.05	1.18 ± 0.06
M2_30_D1			Defect 1		54.05 ± 0.03	1.24 ± 0.08
M2_30_D2			Defect 2		54.02 ± 0.03	1.28 ± 0.07

The cavity diameters were measured thrice per cavity at every 120 degrees in all three cavities of each cavity type and density combination with a caliper gauge.

**Table 2:** Tabular listing of the *p*-values (Welch’s ANOVA with Games–Howell post-hoc test) determined for the parameter Moment M for the various combinations of foam density, cavity type, and test method. The values of *p* < 0.05 were defined as significant. The group of the intact cavity and 30 pcf foam density for Method 2 was excluded, because the group consisted of only one value.

Method	Cavity Type	Foam Density	Lever Out							Edge Load			
			Intact		Defect 1		Defect 2		Intact	Defect 1		Defect 2	
			15 pcf	30 pcf	15 pcf	30 pcf	15 pcf	30 pcf	15 pcf	15 pcf	30 pcf	15 pcf	30 pcf
Lever Out (Method 1)	Intact	15 pcf	-	-	-	-	-	-	-	-	-	-	-
		30 pcf	<0.0001	-	-	-	-	-	-	-	-	-	-
	Defect 1	15 pcf	0.0002	-	-	-	-	-	-	-	-	-	-
		30 pcf	-	0.0052	0.0225	-	-	-	-	-	-	-	-
	Defect 2	15 pcf	0.0001	-	0.2841	-	-	-	-	-	-	-	-
		30 pcf	-	0.0004	-	0.3287	0.0055	-	-	-	-	-	-
Edge Load (Method 2)	Intact	15 pcf	0.0002	-	-	-	-	-	-	-	-	-	-
	Defect 1	15 pcf	-	-	0.0261	-	-	-	0.1812	-	-	-	-
		30 pcf	-	-	-	0.0049	-	-	-	0.0234	-	-	-
	Defect 2	15 pcf	-	-	-	-	0.0011	-	<0.0001	0.1327	-	-	-
		30 pcf	-	-	-	-	-	0.0001	-	-	0.0338	0.031	-

**Table 3:** Tabular listing of the *p*-values (Welch’s ANOVA with Games–Howell post-hoc test) determined for the parameter Interface stiffness k for the various combinations of foam density, cavity type, and test method. The values of *p* < 0.05 were defined as significant. The group of the intact cavity and 30 pcf foam density for Method 2 was excluded, because the group consisted of only one value.

Method	Cavity Type	Foam Density	Lever Out							Edge Load			
			Intact		Defect 1		Defect 2		Intact	Defect 1		Defect 2	
			15 pcf	30 pcf	15 pcf	30 pcf	15 pcf	30 pcf	15 pcf	15 pcf	30 pcf	15 pcf	30 pcf
Lever Out (Method 1)	Intact	15 pcf	-	-	-	-	-	-	-	-	-	-	-
		30 pcf	0.0277	-	-	-	-	-	-	-	-	-	-
	Defect 1	15 pcf	0.9862	-	-	-	-	-	-	-	-	-	-
		30 pcf	-	0.0162	0.9991	-	-	-	-	-	-	-	-
	Defect 2	15 pcf	0.2369	-	0.6694	-	-	-	-	-	-	-	-
		30 pcf	-	0.0050	-	0.3086	0.0128	-	-	-	-	-	-
Edge Load (Method 2)	Intact	15 pcf	0.1158	-	-	-	-	-	-	-	-	-	-
	Defect 1	15 pcf	-	-	0.0207	-	-	-	0.8395	-	-	-	-
		30 pcf	-	-	-	0.0115	-	-	-	0.0135	-	-	-
	Defect 2	15 pcf	-	-	-	-	0.0132	-	0.2703	0.0844	-	-	-
		30 pcf	-	-	-	-	-	0.0007	-	-	0.0362	0.0066	-
